# Supplementary material for: Study of citizen satisfaction and loyalty in the urban area of Guayaquil: Perspective of the quality of public services applying structural equations
Source: PLoS One. 2022 Feb 17;17(2):e0263331. doi: 10.1371/journal.pone.0263331 (PMC8853569; doi:10.1371/journal.pone.0263331)
Supplement: Attachment — Submitted filename: It is an interesting paper that focuses on the citizens.docx [file pone.0263331.s001.docx]

It is an interesting paper that focuses on the citizens’ satisfaction, the expected quality, and perceived quality. The method and results show credible. However, there are some questions that I think should be stated again.

1、	How is the state of arts of your research issue? There is no related works review.

2、	We have much research about citizens’ perception and satisfaction with urban management. What are your contributions, especially the theoretical contributions? Though you have shown in the section of the introduction, what’s the difference between yours and others?

3、	What’s the meaning of ‘experience in territorial and road planning’? Is it pointed to someone who has participated in the territorial and road planning of the urban?

4、	The part of ‘Multigroup Analysis’ seems no direct relationship with your research. While you just show the multigroup in gender, and how are other groups (education? Work? Income?) So, maybe you can give up this part.
