# Supplementary material for: Study of citizen satisfaction and loyalty in the urban area of Guayaquil: Perspective of the quality of public services applying structural equations
Source: PLoS One. 2022 Feb 17;17(2):e0263331. doi: 10.1371/journal.pone.0263331 (PMC8853569; doi:10.1371/journal.pone.0263331)
Supplement: Attachment — Submitted filename: response to Reviewer 2.docx [file pone.0263331.s002.docx]

**PONE-D-21-28034R1

Study of citizen satisfaction and loyalty in the urban area of guayaquil: perspective of the quality of public services applying structural equations

We've checked your submission and before we can proceed, we need you to address the following issues:

1. Thank you for resubmitting your manuscript. As noted previously, during our internal evaluation of the manuscript, we found significant text overlap between your submission and the following previously published works, some of which you are an author.

-** [**https://www.researchgate.net/publication/351365038_The_cultural_and_heritage_tourist_SEM_analysis_the_case_of_The_Citadel_of_the_Catholic_King**](https://www.researchgate.net/publication/351365038_The_cultural_and_heritage_tourist_SEM_analysis_the_case_of_The_Citadel_of_the_Catholic_King) **We would like to make you aware that copying extracts from previous publications, especially outside the methods section, word-for-word is unacceptable. In addition, the reproduction of text from published reports has implications for the copyright that may apply to the publications.**

*****Please revise the manuscript to rephrase the duplicated text, cite your sources, and provide details as to how the current manuscript advances on previous work. Please note that further consideration is dependent on the submission of a manuscript that addresses these concerns about the overlap in text with published work.***

We will carefully review your manuscript upon resubmission, so please ensure that your revision is thorough.**

Thank you very much for your recommendation, an exhaustive review of the manuscript has been carried out in which the paragraphs that could be in other publications of the authors have been modified.
 **2. Can you please upload an additional copy of your revised manuscript that does not contain any tracked changes or highlighting as your main article file.  This will be used in the production process if your manuscript is accepted.  Please amend the file type for the file showing your changes to Revised Manuscript w/tracked changes. Please follow this link for more information:** [**http://blogs.PLOS.org/everyone/2011/05/10/how-to-submit-your-revised-manuscript/**](http://blogs.plos.org/everyone/2011/05/10/how-to-submit-your-revised-manuscript/)

Final manuscript has been uploaded to the platform without change control or highlighting

**3. Please upload a new copy of all Figures as the detail is not clear. Please follow the link for more information:** [**https://blogs.plos.org/plos/2019/06/looking-good-tips-for-creating-your-plos-figures-graphics/**](https://blogs.plos.org/plos/2019/06/looking-good-tips-for-creating-your-plos-figures-graphics/)

We have upload a new copy of all Figures in format jpg to the platform.
